# Setting the Foundations: Individual Rights, Public Interest, Scientific Research and Biobanking

**DOI:** 10.1007/978-3-030-49388-2_2

**Published:** 2020-05-20

**Authors:** Santa Slokenberga

**Affiliations:** 3 Faculty of Law, Uppsala University, Uppsala, Sweden; 4 Academy of Athens, Biomedical Research Foundation, Athens, Greece; 5 Faculty of Law, Stockholm University, Stockholm, Sweden; Faculty of Law, Uppsala University, Uppsala, Sweden

## Abstract

The principle of conferral tames the EU competence to regulate research in a comprehensive manner, yet furthering research is one of its aspirations. Data protection, however, is an area within which the EU has legislated extensively. During the development of the General Data Protection Regulation (GDPR), an important issue to tackle was how to balance the ambitious EU aspirations and differing stakeholder interests, on the one hand, with limited competences in research regulation, on the other, and how to determine the extent to which data protection could be used as a means to further scientific research in the EU legal order. The outcome is the GDPR multifaceted research regime that sets forth EU policy and opens up for further regulations from the Member States as well as the EU.

The research regime that the GDPR has created poses numerous questions. Key among these is, what are the implications of the operationalisation of Article 89 GDPR in biobanking? This chapter sets out some of the underlying tensions in the area and pins down key conceptual foundations for the book. It provides insights into the EU’s interests in the area of biobanking and maps out central elements of the research regime that has been built within the GDPR. Thereafter, it analyses the key concepts used in the book, including biobank and biobanking, scientific research as undertaken under the GDPR, individual rights and public interest. Lastly, it shares some preliminary reflections as starting points for the analysis to come.

## Introduction

The availability, accessibility, acceptability and quality of medical goods and services are of paramount importance to create conditions under which the highest attainable standard of health can be realised.^1^ In achieving these objectives, scientific research, the development of new medicinal products and devices is crucial. In the long term, personalised medicine bears the potential to deliver important changes in medicine as it offers hope for improving health care while also lowering costs. These advances are difficult to achieve unless solid foundations for biobanks are in place and research is furthered.^2^

When scientific research is conceptualised in terms of human rights, the link between biobanking and the right to enjoy the benefits of scientific progress and its applications emerges.^3^ Even though the content of this right is still to be fully appraised,^4^ it is clear that to enjoy the benefits of scientific progress and its applications, there has to be a benefit in the first place.^5^ Therefore, it is crucial that adequate circumstances are created to enable scientific progress to occur.

A coherent regulatory framework has long been seen as key to furthering scientific research and collaboration, within the EU, between the EU and third countries and among the third countries. As has been pointed out on many occasions,^6^ the regulatory landscape is fragmented and this has been a challenge that needs to be tackled.^7^ The first EU legislation in the area of data protection, the Data Protection Directive, made a considerable contribution to shaping the data protection framework for scientific research. However, through foreseeing considerable room for national regulatory autonomy it created a divergent and fragmented lanscape. As will become apparent in this book, the General Data Protection Regulation (GDPR) does not seem to have a strong potential to rectify these divergences. It also has a predisposition to the fragmentation that stems from its DNA, which has already shown some far-reaching implications.

The aim of this chapter is to set out the conceptual foundations for this book. The hope is that it will provide insights into the EU’s interest in the area of biobanking and map out the research regime that has been built around the GDPR. To do this, it analyses the key concepts used in this book: biobank and biobanking, scientific research as undertaken under the GDPR, individual rights and public interest. Lastly, it shares some preliminary reflections as starting points for the analysis carried out in this book, namely on whether the research regime created within the GDPR, which entails the trade-off between the data subjects’ rights and adequate safeguards, is a means to further scientific research and ensure a high level of personal data protection in the EU legal order, and on the implications of such an approach for researchers, law and policymakers, research funders and other stakeholders.

## EU and Biobanking: Building a Research Regime in the Data Protection Framework?

In Europe, historically, the competence to regulate biomedical research has to a considerable degree been placed at the national level, although often it has been exercised with due regard to the hard and soft law instruments in the international fora.^8^ Except for such areas as clinical trials, in the area of biomedical research the EU has traditionally taken a back seat.^9^ However, in biobanking, research is not merely about research regulation, which embraces such questions as the ethical recruitment of research participants and collection of human biospecimens, but also about data protection, which in the EU legal order is classified as a human right under Article 8 of the Charter of Fundamental Rights of the European Union (CFREU) and an area in which the EU has legislative competence under Article 16 of the Treaty on the Functioning of the European Union (TFEU). Against this backdrop, the GDPR, similarly to some degree to its predecessor the Data Protection Directive, faced a considerable challenge in how to effectively operationalise a fundamental right to data protection and further free movement of personal data whilst also accounting for the limits surrounding its competence in research set forth in Article 4(3) of the TFEU, and simultaneously furthering the EU’s objective of competitiveness in the global arena. Arguably, this tension and the legislator’s approach to tackling it is best captured in Recital 4 of the GDPR where it is explained that ‘[t]he processing of personal data should be designed to serve mankind’, and thereafter elaborated that the non-absolute nature of this right entails necessity to balance it against other rights in a proportional manner. Although some of the rights have been mentioned by way of illustration, neither freedom of sciences as protected under Article 13 CFREU nor health care as safeguarded under Article 35 CFREU is indicated. Nonetheless, as the GDPR scientific research regime structure suggests, these two aspirations are inherent elements of the GDPR.

Generally, for the EU, limitations to its competence have not been an issue. In fact, data protection, similar to other areas such as the framework for in vitro diagnostic medical devices, originated as a policy within the Internal Market.^10^ The factual circumstances were that at the time of the Treaty establishing the European Community the European Community’s general competence to regulate the Internal Market was deployed as a tool to develop policies within the Internal Market.^11^ With the Treaty of Lisbon, the circumstances changed and the data protection policy acquired its own legal basis in the Treaty.

This brief historical insight leads to an obvious question, namely, whether the EU’s competence in the area of data protection is now used to push for policies in the areas where the EU currently lacks the competence to adopt harmonisation measures. It is clear the GDPR establishes a research regime, which to some degree can be seen as research harmonisation through the back door: firstly, intra-EU; but secondly, through the extraterritorial clauses and data transfer rules, so also globally.^12^ Yet, this acknowledgement does not come without a ‘however’. The GDPR is a sector-neutral legislation, but each research field comes with its own history and traditions. For example, the area of medical research has been influenced by the horrors of WWII, and the area of biobanking has faced some initial struggles to depart from the stringent rules surrounding research involving human beings.^13^ More recently, biobanking specific research governance measures have been adopted, such as the (revised) World Medical Association Declaration of Taipei on Ethical Considerations Regarding Health Databases and Biobanks (Taipei Declaration).^14^ In terms of competences, the national legal orders have retained varying degrees, and often these competences have been exercised differently, with due regard to the traditions, historical experiences, societal values and objects of public interest. Respect for this diversity was already afforded under the Data Protection Directive. With this background in mind, even if the EU might have possibly desired a different approach and was to assume the test for the limits of its interventions in the area where it lacks direct legislative competence, as the legislative history of the GDPR shows,^15^ this is neither easy to achieve nor realistic. In fact, awareness of the EU’s weakness in the field and the initially-perceived strength of the Council of Europe was demonstrated by an expert group on the ethical and regulatory challenges of international biobank research set up by the European Commission, where in the report ‘Biobanks for Europe. A Challenge for Governance’ it pointed out that the Council of Europe ‘is in a strong position to develop an additional protocol to the Oviedo [Biomedicine] Convention, specifically on biobanking’.^16^ For reasons that are not widely discussed, but arguably relate to the low ratification levels of the previous Biomedicine Convention protocols, instead of an additional protocol the Council of Europe opted for revising its recommendation in the field.^17^

## Building Blocks of the GDPR and the Research Regime

The GDPR can be said to consist of several interrelated fundamental building blocks: principles, individual rights, responsibilities, and oversight and enforcement which give expression to Article 8 CFREU. The principles seek to ensure that personal data are handled properly. The GDPR delineates obligations of the controllers and processors when processing personal data, empowers the data subjects with rights, not only for them to manage their data but also to ensure bottom-up enforcement, and sets forth rules on oversight and enforcement. In practice, however, the lines between these building blocks are rather blurred and the content of these building blocks allows to pose questions about the exact requirements that are stemming from the GDPR. For example, the obligations of controllers and processors are anchored in the data protection principles, but their exact meaning for scientific research is in some respects is unclear, and the oversight and enforcement closely relate to the responsibilities of controllers and processors set forth in the GDPR as well as the data subject rights.

The research regime, which is in-built in the GDPR and rooted in Article 89 GDPR, rests on these building blocks. In terms of principles, the GDPR enables purpose limitation compatibility, permitting secondary use of previously collected data and the processing of these data for scientific research purposes, and storage limitation compatibility, allowing the data to be stored for longer periods if so necessary for scientific research. Yet, reliance on these principles is surrounded by some ambiguity. For example, generally, the GDPR treats the principles of lawfulness and purpose limitation as two distinct principles. Consequently, one could question, whether or not any reuse of data for scientific research purposes needs to have a separate legal ground. In that regard, recital 50 guides that ‘no legal basis separate from which allowed the collection of the personal data is required’ and it adds that ‘[f]urther processing for ... scientific ... research purposes ... should be considered to be compatible lawful processing operations.’ Despite this guidance from the EU legislator, recently it has been argued that ‘[a]s the recital is not accompanied by a specific provision in the main body of the GDPR, this appears not so much a blanket exemption ... but rather advisory’. Therefore, a suggestion to consider purpose compatibility test set forth in Article 6(4) GDPR before proceeding with scientific research has been put forward.^18^ While this precaution can be understandable in the absence of guidance from the Court of Justice of the European Union (CJEU), which holds the ultimate authority under Article 19(1) Treaty on European Union on ‘ensur[ing] that in the interpretation and application of the Treaties [and by extension, secondary law] the law is observed’, one could also take a different stand. It could be argued that scientific research is ‘inbuilt’ in the lawfulness requirements, but in the cases when the EU or the Member States determine and specify the tasks and purposes for the further processing as guided under recital 50 and set forth in Article 6(2) specific consideration to further processing for scientific research could be given. One could also question how the storage limitation should be operationalized, for example, whether it is enough that a controller has the ambition to process the data for scientific research at some point in the future, or this ambition needs to be more concrete. While it is clear that scientific research should not be a guise for storing personal data for other purposes,^19^ it could be argued that the lawmaker has not put constraints for scientific research, disregarding when the research is carried out. However, to avoid unlimited and uncontrolled storage, the research intention should be genuine and demonstrable.

The GDPR provides the data subjects with several rights, known as individual rights, but at the same time through Article 89 it enables two co-existing avenues of depriving the subjects of these rights if necessary for research: first, one that permits the researchers to invoke the GDPR norms directly for the purposes of a particular project; second, one that requires the Member State national law or EU law to be adopted so that derogations can take place.^20^ Both require an individual assessment to take place on whether in a particular case it can be justified to invoke the derogations. Moreover, both make the derogations possible, subject to the conditions and safeguards referred to in Article 89(1) GDPR. Additionally, although it formally does not belong to the research regime that has been set up around Article 89, extensive derogations from individual rights could also be possible through the application of Article 23. The GDPR does not clearly spell out the interplay between Article 23 and 89, nonetheless one could argue that the nature of Article 23 requires that it is applied in exceptional cases only when other avenues are insufficient. Although it cannot be precluded that it could be relied upon in the context of scientific research, those could be expected to be rather rare occasions.

Additionally, within the research regime as well as outside it, the GDPR puts forward a public interest concept, adding to it different qualifiers in different contexts (see below Sect. 4.3.3). This concept enables the application of different data protection requirements to activities that are carried out in the public interest in comparison with those that are not. Likewise, it enables different treatment of those activities that relate to ‘reasons of important public interest’ in comparison with those activities that relate to public interest only.

Generally, the research support afforded under the principles of lawfulness and the possibility to derogate from data subjects’ rights comes with a number of responsibilities for biobanks and researchers. Apart from such practicalities as case-by-case assessments on the necessity and possibility to invoke these derogations, they have to ensure that ‘appropriate safeguards, in accordance with this Regulation, for the rights and freedoms of the data subject’ are in place.^21^ Article 89(1) GDPR further elaborates that ‘[t]hose safeguards shall ensure that technical and organisational measures are in place in particular in order to ensure respect for the principle of data minimisation’. However, the text of the GDPR is not forthcoming on what these safeguards are apart from pinpointing in Article 89(1) that ‘[t]hose measures may include pseudonymisation provided that those purposes can be fulfilled in that manner’, and unpacking what pseudonymisation is under Article 4(5) GDPR. One could argue that reference to the provisions of the Regulation tames the interpretation of ‘appropriate safeguards’ to those GDPR requirements that the controller or processor shall fulfil for a particular scientific research activity (processing), disregarding whether these requirements are set forth in the GDPR or adopted by the Member States when operationalizing provisions of the GDPR, and those that are compatible with the GDPR, for example, because of different scopes of application. However, one can question to what extent they could accommodate safeguards that create obstacles to achieving the GDPR objectives.^22^

Even though the EU is not a research regulator *stricto sensu*, the research regime that is set forth within the GDPR shapes research regulations and thereby practices nationally. To some countries, it may even act as an incentive to revise their frameworks drafted in the early 2000s with great caution vis-à-vis the developments in science and technology. As for countries where biobank legal frameworks have been absent, it can act as an incentive to develop them. However, at the same time it should be kept in mind that although biobanking is an important area, it is only one of the many that a general data protection framework such as the GDPR captures, and that the GDPR in itself cannot be expected to function as the sole base of a research regime for the EU.

## Clarifying Key Concepts and Definitions

### Concepts of Interest

To create a deeper understanding of how Article 89 GDPR has been operationalised in biobank research, it is necessary to pin down two essentials: first, the concept of a biobank and biobanking; second, the approach to individual rights and public interest under the GDPR and within this book.

### Biobank and Biobanking

Biobanks are extensively discussed by scholars as well as law and policy makers, and they are surrounded by a thick layer of governance and regulatory frameworks—hard and soft law measures—but they lack a universally agreed definition. Moreover, sometimes more than one term is used to refer to biobanks, for example, biorepositories and biological resource centres,^23^ and sometimes a distinction between the two is drawn.^24^

Arguably, the term was first used in 1996 and at that time it was mainly used to refer to human population-based biobanks,^25^ despite the fact that collections were being stored at various hospitals and academic institutions even before that time. Moreover, it was a considerable time after the first paraffin embedded tissue sample collections had emerged, which are regarded as ‘the predecessors of today’s biobanks’.^26^

Among law and policy makers, as well as in the literature, a range of definitions can be found.^27^ For example, the 2006 OECD report ‘Creation and Governance of Human Genetic Research Databases’ referred to a biobank as follows: ‘a collection of biological material and the associated data and information stored in an organised system, for a population or a large subset of a population’. However, already in 2009 in the OECD Recommendation on Human Biobanks and Genetic Research Databases, human biobanks and genetic research databases were described as ‘structured resources that can be used for the purpose of genetic research, and which include: (a) human biological materials and/or information generated from their analysis; and (b) extensive associated information’.^28^ This clearly shows the shift from the early focus on a population scale biobank to a more inclusive approach.

Nationally, diverse uses of biobank terminology have appeared. For example, the Swedish Biobanks in Medical Care Act defines a biobank as ‘[b]iological material from one or more human beings that is collected and preserved for an indefinite period, and whose origin is traceable to an individual or individuals’.^29^ The Latvian Human Genome Research Law does not define a biobank but uses the term genome database to refer to what in other countries could be understood as a biobank. In particular, it describes it as ‘a set of data containing coded descriptions of the DNA, coded descriptions of the state of health, coded genealogical and genetic data, as well as coded DNA samples and coded tissue samples to be used for genetic research’.^30^

In practice, however, there is a considerable variation in the types of biobank and their purpose. The term biobank has now commonly been applied not only to refer to human specimen collections but also to plant, animal or microbial samples.^31^ In regard to human biospecimen biobanks, several types can be identified and they can be classified differently.^32^ For example, Harris et al. classify four types, namely: (1) biobanks established as part of the health care process; (2) biobanks established in the context of clinical trials; (3) biobanks comprising specific research project sample collections that can be re-used for other research; and (4) population-based biobanks, which may have a more general research purpose.^33^

Apart from shifts in the content of the biobank concept and the emergence of research data banks (collections of data for further research), changes have occurred in regard to infrastructures and operational management governance. In the early days of biobanking, it was common for record keeping to be confined to a laboratory notebook and specimen storage was in a small number of ultra-low freezers. This is what De Souza and Greenspan describe as a ‘modest style of banking’. Biobanking and its associated science has become a far more complex enterprise.^34^ Driven by technological advances such as automation and computerisation, the management of biobanks has been modernised. Today, specimen annotation and storage location are maintained through electronic records in databases, with the tracking of samples done via a laboratory information management system (LIMS).^35^ Moreover, various software solutions, including with robotic elements, are available and these support biobanks in administrative as well as research practices.^36^ There is also software associated with processes that integrate with LIMS and catalogues of available specimens for an external audience. In the last decade, virtual biobanks have become common,^37^ allowing for easier and faster biospecimen and data transfer and exchange in comparison with centralized model biobanks.^38^ In terms of infrastructure network, BBMRI-ERIC became an important initiative as it created a pan-European directory of biobanks and collection sites that has brought together stakeholders in the field.^39^

For the purposes of this book, given the differences in approaches and lack of universally agreed definition, a broad and inclusive approach to a biobank has been chosen, viewing it as a collection of biospecimens and associated data, including clinical and sample data. The primary focus has been on research biobanks. This approach is in line with what, according to Shaw et al., are seen among the stakeholders as ‘the basic requirements for a biobank’.^40^ By approaching biobanks in such a broad way, the size of a biobank has been rejected as an area of concern. A biobank can be a valuable resource, even without containing a large number of specimens or particularly detailed associated data.^41^

In addition to ‘biobank’, the term ‘biobanking’ is also regularly used in this book. Biobanking involves multiple steps. According to De Souza, with some simplification, they can be expressed in three steps: the collection of a specimen and data, biospecimen processing and storage, and biospecimen dissemination.^42^ This approach was also confirmed in later studies, for example, by Hewitt and Watson.^43^ Therefore, for the purposes of this book, the term has been applied to refer to ‘the collection, processing and storage’ of a specimen and associated data.

### Scientific Research, Individual Rights and Public Interest Under the GDPR and Implications

#### Scientific Research

Although the GDPR establishes a scientific research regime, it does not exhaustively define what scientific research is. In line with guidance provided by the EU legislature in Recital 159,^44^ research can encompass a wide array of activities. It emphasises that ‘the processing of personal data for scientific research purposes should be interpreted in a broad manner including, for example, technological development and demonstration, fundamental research, applied research and privately funded research’. The Article 29 Working Party has indicated that it ‘considers the notion may not be stretched beyond its common meaning and understands that “scientific research” in this context means a research project set up in accordance with relevant sector-related methodological and ethical standards, in conformity with good practice’.^45^ This view is now accepted by the European Data Protection Board.^46^ From this it follows that research within the meaning of the GDPR, albeit on the surface appearing open to interpretation, in fact could be a type of research that follows the requirements of a particular research field.

Recently, the European Data Protection Supervisor, an actor that has been established under another regulation and is tasked to act in regard to personal data protection matters by EU institutions and bodies,^47^ has gone even further and in addition to indicating the importance that ‘relevant sectorial standards of methodology and ethics apply’ for the processing of ‘personal data’ has added that in order scientific research can benefit from the GDPR research regime, ‘the research ... [needs to be] carried out with the aim of growing society’s collective knowledge and wellbeing, as opposed to serving primarily one or several private interests.’^48^ Putting aside the question of the (vague) authority of this actor on the GDPR matters and the fact that the released document is a preliminary opinion only, it suffices to note that although for many reasons it might be appealing to draw a distinction between ‘collective knowledge and well-being’ and ‘primarily one or several private interests’, there are several problems with such an approach. They include uncertainty and ambiguity of the content of these elements and interplay, lack of adequate consideration for the complex reality in which scientific research takes place and commercialization as means to drive the scientific advances forward (e.g. in the area of medicinal products for paediatric use). As derives from the explanations relating to CFREU, Article 13 that protects scientific research relates to Article 10 of European Convention on Human Rights (ECHR), which is not an absolute right. It can be restricted to protect other rights, including privacy (and thereby data protection) of the data subjects under Article 8 ECHR. At the same time, also Article 8 does not contain an absolute right and could be restricted for a number of grounds, including, the economic well-being of the country, the protection of health or morals, or for the protection of the rights and freedoms of others. From such a perspective, a complex balancing act between privacy protection and freedom of expression needs to be exercised, which has strong parallels to that, which is set forth in Article 52(1) CFREU. While carrying out this exercise is beyond the scope of this contribution, it is clear that it should not lead to depriving the data subject of her rights with no (public good) in return and in that way become carte blanche approach to defining scientific research. From such a perspective, one could agree with the Supervisor on the benefits that the research should deliver,^49^ adding that this notion should be generously interpreted. However, it could be argued that the contrast element (‘primarily one or several private interests’) could be difficult to uphold due to the reasons for and the reality in which scientific research is carried out. One can understand that the Supervisor has drawn inspiration from different sources and areas, including the field of copyright, and reasons for doing that, however, one should not be ignorant to the fact that each area comes with its principles that might not necessarily be easily transferable to another field, such as data protection. Finally, although the proposal to defining scientific research that has been put forward by the Supervisor on the surface resonates with the CJEU long-established approach in defining exceptions to rules narrowly, it does not sit well with the legislator’s intention for the field expressed in recital 159 that ‘the processing of personal data for scientific research purposes should be interpreted in a broad manner’. One can only question what reasons should emerge for the CJEU to disregard the signals provided by the legislator for interpreting the text of the GDPR. Acknowledging the complex reality that this uncertainty could create and need for further inquiries, this book proceeds on the assumption that biobanking has a great potential to benefit from the GDPR research regime, disregarding whether or not the Supervisor’s approach is upheld and followed.

#### Individual Rights

A key requirement in biobanking is safeguarding trust. Usually this is achieved through various protections, and is often also expressed in terms of rights of the research participants.^50^ The GDPR does not ignore the rights of individuals and in Chapter III GDPR sets forth a range of data subject rights, in particular the right to information, and it gives further modalities depending on whether or not data are collected directly from the data subject in Articles 13 and 14 respectively. It also provides a right of access under Article 15, a right to rectification under Article 16, a right to erasure under Article 17, a right to restrict processing under Article 18, a right to data portability under Article 20, as well as a right to object and a right not to be subjected to automated decision-making under Article 21. Moreover, Article 19 contains the so-called notification entitlement, whereby a data subject can request to be informed about recipients to whom Article 19 applies.^51^ However, unlike in the human rights discourse and research regulations, under the GDPR self-determination exercised through informed consent is not a right per se but a means to fulfil the lawfulness requirement and could also be seen as a type of adequate safeguards under Article 89(1). The importance of these rights is significant as a means of empowering research participants as data subjects and enabling obstacles related to participants that hold back the work of biobanking to be overcome. On the other hand, in some cases these very same rights can also hinder research if they are exercised. To overcome this, the GDPR sets forth the already-noted derogation mechanism, which has previously been characterised as a mechanism that strips individuals of their rights.^52^

#### Public Interest

There are different approaches how to approach the notion of public interest. A theory of public interest has been conceptualized as ‘the process of defining the scope of rights and the justification for securing public goods as the objects of collective rights’.^53^ However, the GDPR seems to depart from this complex public good and public interest tangle and takes a more practical approach. It approaches public interest as an end in itself, allowing for additional regulatory privileges. As highlighted below, this usually comes at the expense of individual rights, but is not necessarily limited to that. Hence, more broadly under the GDPR public interest can be described as an object worth safeguarding for the needs or interests of the Member States or the EU for the purposes of which a number of specific measures could be taken, including the rights of a data subject could be constrained.

In relation to biobanking and public interest a number of questions emerge. One can discuss, under what circumstances, if at all, is biobanking a public interest. One can also question, whether there is a difference for what purpose research is conducted and who the researcher or research institution. For example, whether it is a non-profit actor carrying out research in the area of non-communicable diseases, which is a large cause of death across the world,^54^ or commercially-driven research relating to the identification of genes attributed to traits or a child’s potential talent. If so, who is the one to decide?

In the GDPR, public interest is mentioned 70 times, yet on none of these occasions is the concept fully explained. Moreover, qualifiers can be found, for example, Recital 50 refers to the ‘general public interest’, Recital 70 to ‘important objectives of general public interest’, Recital 112 to ‘important reasons of public interest’ and Article 18(2) GDPR to ‘reasons of important public interest’. In spite of this, a number of clues can be found that indicate that these qualifiers have different meanings. Therefore, while as guided by Recital 159 research in the area of public health could be located in the area of public interest in some situations, this very same research might not necessarily benefit from laxed measures applicable to activities falling under ‘important reasons of public interest’.

Perhaps the most central operationalisation of public interest relates to the lawful processing of personal data. It can be derived from Articles 6(2) and 6(3) GDPR that research can be considered by a Member State to be in the Member State’s public interest.^55^ Moreover, for the purposes of tasks carried out in the public interest, the implicit prohibition on the processing of personal data can be lifted.^56^ This possibility has to be further regulated by EU law or Member State national law.^57^ One could say that by using the open-ended concept of public interest, the GDPR allows Member States to choose their own policies. As mapped out by Reichel and Lind, in the earlier drafts of the GDPR it was suggested that the Commission should define the concept of public interest (at that time, ‘high public interest’). This was heavily criticised since it would de facto mean that the Commission could control the Member States in areas that were politically sensitive.^58^ Hence, this approach was not retained in the GDPR. Therefore, Member States could decide that, for example, tackling Covid-19 or the development of personalised medicine are matters of public interest. However, that in itself would not be sufficient to proceed with the processing of personal data as other requirements, including those set forth in Article 9 also shall be met.

#### Interaction Between Scientific Research, Individual Rights and Public Interest

On a number of occasions in the GDPR public interest coexists with the research regulatory framework for individual rights. However, for example, under Article 17(3) the two are addressed differently. Article 18(2) GDPR *expressis verbis* relates to ‘reasons of important public interest of the Union or of a Member State’, which may well be research. Similarly, also Article 20(3) refers to ‘the performance of a task carried out in the public interest’, but does not in itself contain provisions relating to research. This differentiation is also present in Article 21(6) GDPR, which merges these two regimes, the research and the public interest. Under Article 21(1) GDPR, ‘[t]he data subject shall have the right to object, on grounds relating to his or her particular situation, at any time to processing of personal data concerning him or her which is based on point (e) or (f) of Article 6(1), including profiling based on those provisions’. In accordance with Article 21(6) GDPR, ‘[w]here personal data are processed for scientific (...) research purposes (...) pursuant to Article 89(1), the data subject, on grounds relating to his or her particular situation, shall have the right to object to processing of personal data concerning him or her, unless the processing is necessary for the performance of a task carried out for reasons of public interest’. In that way, the operational scope of the right to object is restricted when research is carried out in the public interest.

However, this public interest interplay with research regulation has to be characterised even more specifically. Article 89(2) GDPR permits derogations from individual rights for Articles 15, 16, 18 and 21 GDPR. In that way, research in the public interest in comparison with research not falling in the public interest benefits from an Article 20 and Article 21 derogation.

Furthermore, apart from these avenues, Article 23 GDPR is of interest. Article 23(1) GDPR states that ‘Union or Member State law to which the data controller or processor is subject may restrict by way of a legislative measure the scope of the obligations and rights provided for in Articles 12 to 22 and Article 34, as well as Article 5 in so far as its provisions correspond to the rights and obligations provided for in Articles 12 to 22, when such a restriction respects the essence of the fundamental rights and freedoms and is a necessary and proportionate measure in a democratic society to safeguard ‘(e) other important objectives of general public interest of the Union or of a Member State, in particular (..) public health (..)’. It cannot be excluded that there could be a possibility for the Member States to rely on this provision for particular research purposes.

There is a rather subtle difference in terms of individual rights for how a Member State approaches research, and whether and to what extent it locates it in the area of public interest. However, for obligations stemming from the GDPR,^59^ as well as data transfer to third countries and international organisations, public interest conceptualization has a considerable role to play.^60^ Nevertheless, as this book will show, there are Member States that have not afforded any particular consideration to research being or not being in the public interest within the GDPR. Moreover, this term is occasionally used interchangeably with ‘public goods’—in this way explaining to what extent, if at all, biobanking is seen as an interest worth safeguarding and what means are used to further this interest.

#### Implications

It is rather clear that theoretically permissible differences between the level of protection in different EU Member States should not become an obstacle to free movement of personal data. It could, however, be different in practice. One could also question, to what extent, if at all, could forum shopping take place? Arguably, the most relevant guidance on the question of choice of jurisdiction may be inferred from the *Weltimmo* case in which the place of establishment of a controller was emphasized.^61^ However, that establishment is subject ‘to any real and effective activity—even a minimal one—exercised through stable arrangements’.^62^ This very same approach is now specified in Recital 22 of the GDPR, though without the requirement of ‘even a minimal one’.^63^ It is unclear yet whether absence of the indication of this minimum threshold will have any practical significance under the GDPR.

In practice, for collaborative research projects, as long as the real and effective activity requirement exercised through the stable arrangements requirement can be met, then forum shopping could take place. For this, private international law could, to some degree, become handy. Yet, what is the practical significance of this forum shopping is another question to ask as the research ethics committees are not necessarily required to approve lawful research that appears unethical.^64^ On the other hand, ethics is not necessarily ethics only (not binding, but highly recommended). Often it is a legal requirement to receive an ethics review and the research ethics committees operate under a legal framework. It may well happen that the research ethics committee’s decision becomes an obstacle to free movement of personal data in scientific research, and then it could ultimately be for the CJEU to address it and contextualize in relation to the GDPR. If ethical approval is treated as safeguards, then indeed, such an obstacle could be justified. However, if the wording in Article 89(1) ‘in accordance with this regulation’ applies only to measures under the GDPR stricto sensu, one could question whether the approach taken by Article 29 Working Party can be upheld. As the CJEU has demonstrated in a different context, it is willing to accommodate genuine ethics concerns even when the legislator has not done that in a clear manner,^65^ and therefore it could be argued that a similar approach could also be taken under the GDPR.

## Concluding Remarks

Concerns over the restrictive approach to data protection were expressed when the Commission’s initial text was negotiated in the legislative procedure.^66^ In particular, there were concerns that the draft GDPR, if adopted, may ‘challenge the survival of retrospective clinical research, biobanking, and population-based cancer registries in the EU’^67^ and over whether the trilogue—key players in the EU ordinary legislative procedure (the Commission, the European Parliament and the Council)—would accept the importance of health research and would not hinder it.^68^

The text of the GDPR as adopted and applicable continues to raise concerns. For the law and policy makers, it opens up room for considerable variation in how data protection is further regulated nationally. For researchers and biobankers, it raises questions on compliance with the rules of the GDPR as invoked directly and further specified nationally when carrying out research. For the data subjects, it raises questions of the level of protection the GDPR provides them and on the meaning of the fundamental right to the protection of personal data as safeguarded under Article 8 CFREU. As Pormeister questions, does the GDPR go too far?^69^ Staunton et al. also implicitly point in that direction as they agree that the GDPR is stripping data subjects of their rights,^70^ but this does not necessarily mean that no protection has been afforded to the data subjects. The limitations to individual rights are prescribed at the expense of appropriate safeguards, to ensure that a high level of protection of personal data is not undermined. Therefore, it is important that these safeguards are fully operationalized and a fair balance between valid objectives, in particular data privacy protection and scientific research, is struck.

However, in the case of biobanking and from the perspective of the GDPR, it is the Member States who have the ultimate say whether the flexibility that the GDPR offers could and should be used with due regard to their particular circumstances, such as history, traditions, cultural values and prevailing views in society. Whether the stakeholders will manage to reconcile these divergences with a view to further research through the elaboration and adoption of a code of conduct in the field pursued by BBMRI-ERIC remains to be seen.^71^ One could call such a task ambitious as the stakeholders through the code of conduct are attempting to resolve this when the trilogue together with the Member States could not do so during the legislative procedure.

## Footnotes

1 See ICESCR Article 12 and General Comment No. 14.E/C.12/2000/4 (2000).

2 Hewitt (2011), pp. 112–119.

3 As a human right, it is set forth in Article 27.1 of the Universal Declaration of Human Rights and Article 15 of the International Covenant of Economic, Social and Cultural Rights. Article 27.1 of UDHR states that ‘[e]veryone has the right freely... to share in scientific advancement and its benefits’. In a similar vein, Article 15.1.b ICESCR states that ‘[t]he States Parties to the present Covenant recognize the right of everyone:... [t]o enjoy the benefits of scientific progress and its applications’.

4 Among most recent contributions see Committee on Economic, Social and Cultural Rights, General comment No. 25 (2020) on science and economic, social and cultural rights (article 15 (1) (b), (2), (3) and (4) of the International Covenant on Economic, Social and Cultural Rights),E/C.12/GC/25.

5 Slokenberga and Howard (2019).

6 See, for example, Directorate-General for Research and Innovation (European Commission) (2012), pp. 46–48.

7 See, for example, Chen and Pang (2014), pp. 113–117. Furthermore, biobank governance remains also a regional challenge Kaye (2006), pp. 245–248. In that regard, solutions have also been sought, among which there is the Code of Conduct for international genomic research. See Knoppers et al. (2011).

8 For example, Council of Europe treaties, such as Convention for the protection of Human Rights and Dignity of the Human Being with regard to the Application of Biology and Medicine: Convention on Human Rights and Biomedicine and its Additional Protocol to the Convention, concerning Biomedical Research, various recommendations in the field, as well as WMA Helsinki declaration, and CIOMS International Ethical Guidelines for Health-related Research Involving Humans.

9 This, however, is undergoing changes. The *In Vitro Diagnostic Medical Devices Regulation* (applicable from 2022) contains provisions relevant to biobanking.

10 Slokenberga (2016), ch. 6.2.3.3.

11 De Witte (2006).

12 Slokenberga et al. (2019), pp. 30–48.

13 Stjernschantz Forsberg (2012).

14 World Medical Association (2016).

15 See Reichel J, Lind A-S (2015) The new general data protection regulation—where are we are and where might we be heading? In: Mascalzoni D (ed) Ethics, law and governance of biobanking: national, European and international approaches. Springer, Dordrecht, pp 95–100.

16 Directorate General for Research and Innovation (2012), p.47.

17 See Recommendation CM/Rec(2016)6 of the Committee of Ministers to member States on research on biological materials of human origin (Adopted by the Committee of Ministers on 11 May 2016 at the 1256th meeting of the Ministers’ Deputies).

18 See European Data Protection Supervisor, A Preliminary Opinion on data protection and scientific research, 6 January 2020, pp. 20–21. Such a cautions approach has also been flagged by scholars, for example Bell et al. (2019), pp. 43–53, at 48.  

19 European Data Protection Supervisor, A Preliminary Opinion on data protection and scientific research, 6 January 2020, pp. 23–24.

20 While this assessment is intended to be case-by-case-based in accordance with the wording of the GDPR, as the analysis carried out by Tzortzatou et al. show some Member States opt for generic derogations, potentially leaving room for further specification in their national, biobank-specific laws.

21 Article 89(1) GDPR. See Anne-Marie Duguet Jean Herveg ‘Safeguards and derogations relating to processing for scientific purposes: Article 89 analysis for biobank research’ in this book.

22 See further analysis on appropriate safeguards by Anne-Marie Duguet Jean Herveg ‘Safeguards and derogations relating to processing for scientific purposes: Article 89 analysis for biobank research’ in this book.

23 Parodi (2015).

24 See, for example, Siwek (2015).

25 Hewitt and Watson (2013), p. 309.

26 Biobanking and Biomolecular Resources Research Infrastructure (2013).

27 Shaw et al. (2014), pp. 223, 226.

28 OECD (2009), p. 22.

29 Sveriges Riksdag (2002), Chapter 1 Section 2.

30 LR Saeima (2002), Section 1 Subparagraph 8.

31 Hewitt and Peter (2013), pp. 309, 313.

32 EU Commission (2012), pp. 14–17.

33 Harris et al. (2012).

34 De Souza and Greenspan (2013).

35 See Bendou et al. (2017).

36 De Souza and Greenspan (2013). For a more detailed insight, see Müller et al. (2017).

37 Reijs et al. (2015).

38 Somiari and Somiari (2015), pp. 12–27, at 19

39 BBMRI-ERIC http://www.bbmri-eric.eu/.

40 Shaw et al. (2014), p. 226. These seem to be shared in a study by Hewitt and Watson, Defining Biobank. Additionally, they point at the importance of managing biobanks according to professional standards. Hewitt and Watson (2013), pp. 309, 313. As this is a governance question rather than directly related to individual rights, we have ommitted this criterion from the approach.

41 Shaw et al. (2014), p. 227.

42 De Souza and Greenspan (2013).

43 Hewitt and Watson (2013), p. 311.

44 It states ‘[w]here personal data are processed for scientific research purposes, this Regulation should also apply to that processing. For the purposes of this Regulation, the processing of personal data for scientific research purposes should be interpreted in a broad manner including for example technological development and demonstration, fundamental research, applied research and privately funded research. In addition, it should take into account the Union’s objective under Article 179(1) TFEU of achieving a European Research Area. Scientific research purposes should also include studies conducted in the public interest in the area of public health. To meet the specificities of processing personal data for scientific research purposes, specific conditions should apply in particular as regards the publication or otherwise disclosure of personal data in the context of scientific research purposes. If the result of scientific research in particular in the health context gives reason for further measures in the interest of the data subject, the general rules of this Regulation should apply in view of those measures.’

45 EU Article 29 Working Party Guidelines on consent under Regulation 2016/679 (2017), pp. 27–28.

46 See the European Data Protection Board, Endorsement 1/2018.

47 Regulation (EU) 2018/1725 of the European Parliament and of the Council of 23 October 2018 on the protection of natural persons with regard to the processing of personal data by the Union institutions, bodies, offices and agencies and on the free movement of such data, and repealing Regulation (EC) No 45/2001 and Decision No 1247/2002/EC, OJ L 295, 21.11.2018, pp. 39–98, Article 52.2.

48 European Data Protection Supervisor, A Preliminary Opinion on data protection and scientific research, 6 January 2020, p. 12.

49 European Data Protection Supervisor, A Preliminary Opinion on data protection and scientific research, 6 January 2020, p. 12.

50 For an overview, see Staunton et al. (2019).

51 Notification obligation regarding rectification or erasure of personal data or restriction of processing. The controller shall communicate any rectification or erasure of personal data or restriction of processing carried out in accordance with Article 16, Article 17(1) and Article 18 to each recipient to whom the personal data have been disclosed, unless this proves impossible or involves disproportionate effort. The controller shall inform the data subject about those recipients if the data subject requests it.

52 Staunton et al. (2019).

53 Capps (2012), p. 240.

54 World Health Organization (2018).

55 Recital 45 guides that ‘[i]t should also be for Union or Member State law to determine whether the controller performing a task carried out in the public interest or in the exercise of official authority should be a public authority or another natural or legal person governed by public law, or, where it is in the public interest to do so, including for health purposes such as public health and social protection and the management of health care services, by private law, such as a professional association.’

56 Recital 10, Article 6(1)(f) and 6(2) GDPR.

57 As clarified in Recital 45, ‘[t]his Regulation does not require a specific law for each individual processing. A law as a basis for several processing operations based on a legal obligation to which the controller is subject or where processing is necessary for the performance of a task carried out in the public interest or in the exercise of an official authority may be sufficient.’

58 Reichel and Lind (2015), pp. 95–100.

59 For example, regarding processors under Article 28(3)(a), regarding data protection impact assessment under Article 35(9), in regard to a prior consultation under Article 36(5) GDPR.

60 Recital115, Article 49 GDPR. See further, the European Data Protection Board (2018), pp. 10–11.

61 Weltimmo s. r. o. V Nemzeti Adatvédelmi és Információszabadság Hatóság, ECLI:EU:C:2015:639, para 24. For a discussion on territoriality under the Data Protection Directive see Maja Brkan (2016). For insights under the GDPR see Pormeister (2018).

62 Court of Justice of the European Union, Weltimmo s. r. o. V Nemzeti Adatvédelmi és Információszabadság Hatóság, ECLI:EU:C:2015:639, para 31.

63 Recital 22, GDPR.

64 See also Article WP 29 (2017).

65 See Case C-165/08, Commission of the European Communities v Republic of Poland, ECLI:EU:C:2009:473.

66 Gottweis et al. (2012).

67 Kerr (2014), p. 563.

68 Coppen et al. (2015), p. 757.

69 Pormeister (2017), pp. 137–146.

70 Staunton et al. (2019).

71 See http://code-of-conduct-for-health-research.eu/.
